# Bet Hedging to Aid Seed-Based Wetland Restoration Under Hydrologic Extremes

**DOI:** 10.1007/s13157-025-01935-7

**Published:** 2025-05-22

**Authors:** Elana V. Feldman, Karin M. Kettenring

**Affiliations:** Department of Watershed Sciences and Ecology Center, Utah State University, Logan, UT USA

**Keywords:** Functional diversity, Hydrology, Invasion resistance, Invasive plant management, Native plants, Native seeding density, *Phragmites australis*, Seed sowing, Wetland revegetation

## Abstract

Reestablishing native plant communities following invasive species management is a common restoration goal for wetland managers. Although passive recolonization is occasionally sufficient, often, active revegetation through seeding is required. However, the outcomes of seeding likely differ by site (due to varying environmental conditions) and the composition of the seed mix. We evaluated the effects of both passive recolonization and seeding treatments (varying density and diversity of natives) on wetland plant community cover and composition at two sites in the Great Salt Lake Watershed, USA, over two years. We found that passive recolonization was insufficient to increase native plant cover at one location and limit invasive species' cover at either location. Furthermore, different emergent wetland restoration sites, despite geographic proximity, had different plant community outcomes, likely due to distinct site environmental conditions. We also found that the effects of the seeding treatments appeared to be overwhelmed by water depth due to two extreme weather events (severe drought in year 1 and prolonged flooding in year 2). However, these events provided an opportunity to observe the recovery potential of the different functional groups and identify three species of restoration interest (*Bolboschoenus maritimus, Schoenoplectus acutus,* and *Distichlis spicata*) that were able to survive the extreme conditions during both growing seasons at one site. These findings underscore the importance of not relying on passive recolonization and instead using bet-hedging strategies (e.g., seeding diverse mixes with species with a range of hydrologic tolerances) to overcome hydrologic extremes, conditions likely to become more common with climate change and ever-increasing upstream water diversions.

**Supplementary Information:**

The online version contains supplementary material available at 10.1007/s13157-025-01935-7.

## Introduction

Reestablishing diverse, native wetland plant communities is a common restoration goal to recover lost wildlife habitat and other aspects of ecosystem functioning (Moreno-Mateos et al. 2012; Davidson 2014). Occasionally, in wetland restoration, the seed bank of the restoration site is sufficient to support native recovery. However, in sites with a long history of plant invasions where seed banks are depauperate, revegetation is an essential step to both increase native plant cover and minimize reinvasion and secondary invasions following invasive species management (Adams and Galatowitsch 2008; Kettenring and Adams 2011). Nonetheless, active revegetation (seeding; planting) is not always pursued. Wetland revegetation can be expensive and logistically complicated (Kettenring and Tarsa 2020), diverse seed supplies may be unavailable (Henry et al. 2024a), and restoration practitioners may lack the expertise to successfully seed and plant (Henry et al. 2024b). Furthermore, the high mortality of seeds and seedlings in restorations (Bohnen and Galatowitsch 2005; Larson et al. 2015) raises questions about the value of seeding, especially as restoration conditions become more extreme with climate change. There are uncertainties about when seeding is necessary in wetlands, whether it improves restoration outcomes, and how best to design seed mixes to achieve goals.

When seeding is implemented, two factors that can be altered by restoration practitioners to influence plant community cover and composition (the number and identity of species that are present) are density and functional diversity (Maron and Marler 2007; Kettenring and Tarsa 2020; Robinson et al. 2024a). High native seeding densities can increase both the cover of sown species and the chance that less competitive species will emerge even in the face of high seed and seedling mortality (Wilkerson et al. 2014; Grman et al. 2020; Tarsa et al. 2022a) while simultaneously suppressing unwanted species (i.e., biotic resistance to invasive species; Byun et al. 2023). Functional diversity, based on the diversity of traits within a community (Funk et al. 2008), can also drive plant community composition through resource competition and niche partitioning (Maron and Marler 2007; Robinson et al. 2024a). For example, species that are part of the forb functional group can grow quickly and preempt resources from an invasive grass, maintaining native plant communities by reducing invader growth (Byun et al. 2020; Robinson et al. 2024a). Altering these two factors can increase the success of a restoration project by enhancing native plant communities and their resistance to invasives, although it is unclear how these factors will respond under different conditions, especially as conditions become more extreme with climate change.

To develop generalizable results from restoration experiments, treatments should be tested at multiple sites with potentially different environmental conditions. Both establishment and biotic resistance of native plants can be altered in geographically proximate sites due to abiotic conditions (such as salinity and hydrology) and biotic conditions (e.g., differing levels of competition from invasives; Young et al. 2015; Fried et al. 2018). For example, even for species adapted to local site conditions, germination from the seed bank can be inhibited by factors such as flooding (Fraser et al. 2014) and long-term water-level fluctuations (Wilcox and Nichols 2008). Understanding how the effects of seeding treatments differ between sites, and therefore which ones are generalizable across locations, is critical for restoration practitioners designing plans for revegetation.

We were interested in determining the effect of passive recolonization and seeding on wetland community cover and composition at two locations in the Great Salt Lake Watershed, USA, over two growing seasons. We asked four questions: (1) What species recolonize passively from the seed bank following invasive species management? We predicted that passively emerging species cover would be low for native species due to a depleted seed bank from years of invasive species presence and low for invasive species following intensive removal of the invasive species by land managers (Rohal et al. 2021). (2) Does seeding alter wetland plant community cover and composition? We predicted that seeded plots would have different species and a higher native cover than unseeded plots as well as lower invasive species cover due to the invasion resistance of the seeded native community (Adams and Galatowitsch 2008; Tarsa et al. 2022a) (3) When seeding, how does altering seed mix functional group identity and density affect plant community cover and composition? We predicted that a greater seed mix density would result in a greater number of species and a higher native plant cover (Tarsa et al. 2022a) and that a higher native seeding density and an increased proportion of native forbs would reduce invasive species cover (Byun et al. 2020; Robinson et al. 2024a). (4) Does passive recolonization and the effects of seeding differ between two restoration sites within the same watershed? We predicted that despite the geographic proximity of the two sites, the passively and actively restored plant communities would differ due to divergent site environmental conditions and hydrologic management capabilities (Young et al. 2015; Fried et al. 2018).

## Methods

### Experiment Overview

We explored the effects of native seeding density and functional group identity on seeded, unseeded native, and unseeded invasive plant cover at two locations over two years (2022 and 2023). At each site, we implemented a full factorial experiment examining two levels of density and five levels of functional group identity. Species were planted in mixes of three species with all three species representing the same functional group (Table 1). Functional groups were based on family, longevity (perennial or annual), and growth form (forb or graminoid). Native seeding densities were 1x = 1938 pure live seed (PLS) m^−2^ or 5x = 9688 PLS m^−2^, which were selected based on recommended seeding densities for the region (Tarsa et al. 2022b). We also included an unseeded control to characterize the community that emerged from the seed bank (Table 2).

**Table 1:** Native species in each of the five seed mixes (representing a functional group) with their common names, families, life durations, and wetland indicator statuses

Seed Mixes and Associated Species	Common Name	Plant Family	Duration	Wetland Indicator Status
*Annual Forb Seed Mix*				
*Bidens cernua* L	Nodding beggarstick	Asteraceae	Annual	Obligate
*Rumex maritimus* L	Golden dock	Polygonaceae	Annual	Facultative wetland
*Symphyotrichum ciliatum* (Ledeb.) G. L. Nesom	Rayless alkali aster	Asteraceae	Annual	Facultative wetland
*Bulrush Seed Mix*				
*Bolboschoenus maritimus* (L.) Palla	Alkali bulrush	Cyperaceae	Perennial	Obligate
*Schoenoplectus acutus* (Muhl. ex Bigelow) Á. Löve & D. Löve	Hardstem bulrush	Cyperaceae	Perennial	Obligate
*Schoenoplectus americanus* (Pers.) Volkart ex Schinz & R. Keller	Chairmaker’s bulrush	Cyperaceae	Perennial	Obligate
*Grass Seed Mix*				
*Distichlis spicata* (L.) Greene	Saltgrass	Poaceae	Perennial	Facultative
*Muhlenbergia asperifolia* (Nees & Meyen ex Trin.) Parodi	Scratchgrass	Poaceae	Perennial	Facultative wetland
*Puccinellia nuttalliana* (Schult.) Hitchc	Nuttall’s alkaligrass	Poaceae	Perennial	Facultative wetland
*Perennial Forb Seed Mix*				
*Euthamia occidentalis* Nutt	Western goldentop	Asteraceae	Perennial	Facultative wetland
*Eutrochium maculatum* (L.) E.E. Lamont	Spotted Joe Pye weed	Asteraceae	Perennial	Obligate
*Solidago canadensis* L	Canada goldenrod	Asteraceae	Perennial	Facultative upland
*Rush Seed Mix*				
*Juncus arcticus* Willd	Mountain rush	Juncaceae	Perennial	Obligate
*Juncus gerardii* Loisel	Saltmeadow rush	Juncaceae	Perennial	Facultative wetland
*Juncus torreyi* Coville	Torrey’s rush	Juncaceae	Perennial	Facultative wetland

*Bidens cernua* included the native species *Bidens cernua, Bidens comosa,* and *Bidens frondosa* due to a lack of distinguishing features during collection. Scientific names and wetland indicator status are from the USDA NRCS PLANTS Database (USDA NRCS 2023). Wetland indicator status refers to a species’ likelihood of occurring in a wetland. Possible status includes obligate (almost always in a wetland), facultative wetland (usually in a wetland), facultative (equal chance of wetland versus non-wetland), facultative upland (usually not in a wetland), and upland (almost never in a wetland). Species richness was held constant at three species for each mix. All mixes were sown in equal PLS proportions

**Table 2:** Species that emerged from the seed bank in the field over the course of the experiment and their common names, invasive statuses, wetland indicator statuses, and field site locations (FB = Farmington Bay, UL = Utah Lake)

Species	Common Name	Invasive Status	Wetland Indicator Status	Location
*Alopecurus pratensis* L	Meadow foxtail	Invasive	Facultative wetland	UL
*Asclepias incarnata* L	Swamp milkweed	Native	Obligate	UL
*Bassia scoparia* (L.) A. J. Scott	Burningbush	Invasive	Facultative	UL
*Chenopodium* spp. L	Goosefoot	Native	Facultative	FB, UL
*Cynodon dactylon* (L.) Pers	Bermudagrass	Invasive	Facultative upland	UL
*Cyperus erythrorhizos* Muhl	Redroot flatsedge	Native	Obligate	UL
*Echinochloa crus-galli* (L.) P. Beauv	Barnyardgrass	Invasive	Facultative wetland	UL
*Lactuca serriola* L	Prickly lettuce	Invasive	Facultative upland	UL
*Leptochloa fusca* (L.) Kunth	Malabar sprangletop	Native	Facultative wetland	FB
*Phragmites australis* (Cav.) Trin. ex Steud	Common reed	Invasive	Facultative wetland	FB, UL
*Polygonum persicaria* L	Spotted ladysthumb	Native	Facultative wetland	UL
*Populus fremontii* S. Watson	Fremont cottonwood	Native	Facultative wetland	UL
*Ranunculus cymbalaria* Pursh	Alkali buttercup	Native	Obligate	UL
*Rumex stenophyllus* Ledeb	Narrowleaf dock	Invasive	Facultative wetland	FB
*Salicornia rubra* A. Nelson	Red swampfire	Native	Obligate	FB
*Salix amygdaloides* Andersson	Peachleaf willow	Native	Facultative wetland	UL
*Schoenoplectus pungens* (Vahl) Palla	Common three-square bulrush	Native	Obligate	UL
*Tamarix* spp. L	Tamarisk	Invasive	Facultative	FB, UL
*Typha* spp. L	Cattail	Invasive	Obligate	FB, UL

*Chenopodium*, *Tamarix*, and *Typha* were only identified to genus due to cryptic species that prevent proper identification without additional genetic testing. Scientific names and wetland indicator status are from the USDA NRCS PLANTS Database (USDA NRCS 2023). Wetland indicator status refers to a species’ likelihood of occurring in a wetland. Possible status includes obligate (almost always in a wetland), facultative wetland (usually in a wetland), facultative (equal chance of wetland versus non-wetland), facultative upland (usually not in a wetland), and upland (almost never in a wetland). Invasive status was determined using Downard et al. (2017) and includes both native and non-native species

### Plant Communities

All species seeded in these experiments (Table 1) are native to the study sites in Great Salt Lake and Utah Lake wetlands (Downard et al. 2017) and represent native species of interest for achieving restoration goals (Rohal et al. 2018; Kettenring et al. 2020; Henry et al. 2024b). The native species for these experiments represent a range of durations and hydrologic tolerances (as represented in their wetland indicator status ratings; Table 1) and were selected based on potential performance to reduce invasive species (especially the non-native grass *Phragmites australis*; Rohal et al. 2016, 2019a,b; Hebert 2022; Robinson et al. 2024a; Tarsa et al. 2022a,b).

There are several invasive species common in wetlands in the study region (Kettenring et al. 2020), although *P. australis* is the most problematic and constitutes the majority of weed management budgets (Rohal et al. 2018; Kettenring and Mock 2012; Young and Kettenring 2020). Non-native *P. australis* is a widespread, robust invasive plant that outcompetes native species in wetlands across North America (Saltonstall 2002; Kettenring et al. 2012) and degrades wildlife habitat (Benoit and Askins 1999; Robichaud and Rooney 2017; Whyte et al. 2015)*.* Other invasive species that negatively impact wildlife habitat or are otherwise not desired by managers but with less dramatic effects include *Tamarix* spp., *Typha* spp., other forbs (e.g., *Bassia scoparia*, *Lactuca serriola*, *Rumex stenophyllus*), and various invasive grasses (e.g., *Alopecurus pratensis*, *Cynodon dactylon*, *Echinochloa crus-galli*; Table 2). Given that establishment is a critical period for seedlings of *P. australis* (Byun et al. 2013; Kettenring et al. 2015) and many other species (Schupp 1995; Turnbull et al. 2000), there is keen interest in promoting native plant communities to reduce invasive presence through biotic resistance in wetlands.

### Study Sites

**Fig. 1:**
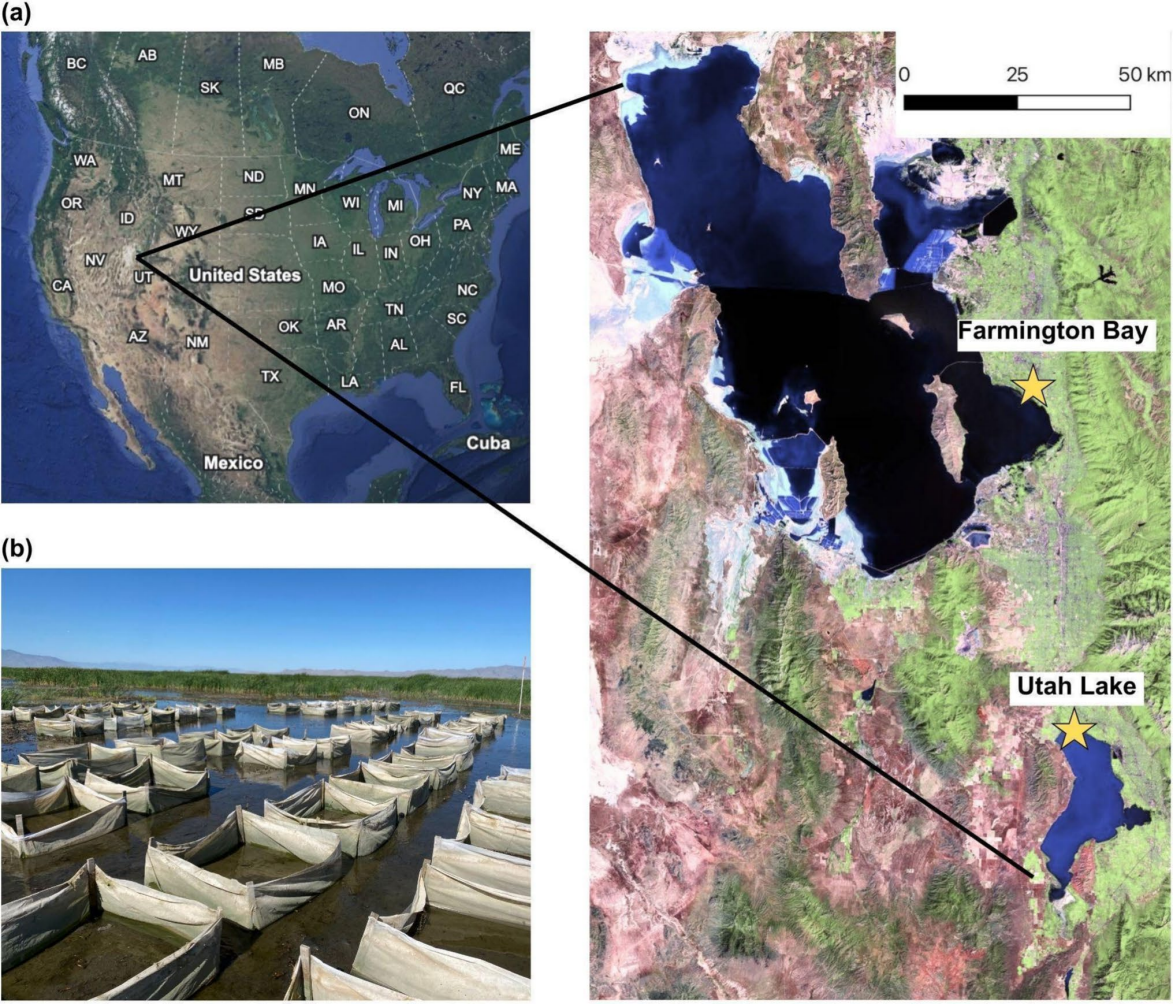
**a** Field site locations at the Farmington Bay of the Great Salt Lake and the north shore of Utah Lake in Utah, USA. **b** Photo of the seeded plots surrounded by mesh fabric, taken at Farmington Bay in July 2022. Map was created with imagery from ETM + Pan Mosaics (1999–2003) and Google Earth 10.49.0.0 (4/10/2013–12/14/2015)

**Fig. 2:**
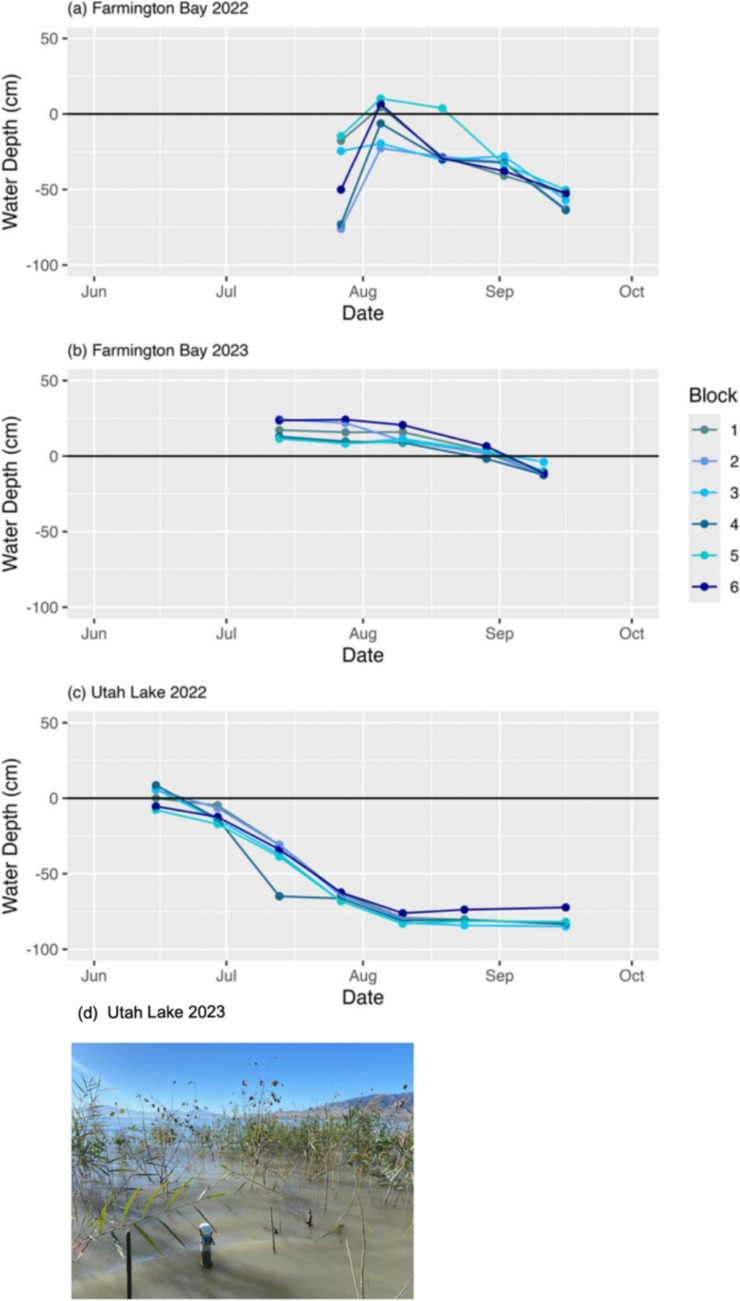
Water depth (cm) in each block on each sampling date at Farmington Bay during (**a**) 2022 and (**b**) 2023 and (**c**) Utah Lake during 2022. The black line at y = 0 indicates the soil surface. The variable start dates for water depth monitoring reflect when the sites could be accessed. **d** Utah Lake was not sampled in 2023 due to plot inaccessibility from extreme flooding; water depth in 2023 was over 1.5 m and the actual plots were inaccessible by foot. The photo here shows a 2 m tall groundwater well from a different experiment in the vicinity (upslope) of the plots from the present study

We conducted this experiment at two locations: Farmington Bay Waterfowl Management Area on the east side of the Great Salt Lake and the north shore of Utah Lake (Fig. 1a; hereafter “Farmington Bay” and “Utah Lake”). Great Salt Lake wetlands, where Farmington Bay is located, is one of the largest wetlands in the western United States and provides critical habitat for migratory birds (Intermountain West Joint Venture 2013). Water levels in many Great Salt Lake wetlands are controlled by managers through a series of dikes and head gates in wetland impoundments, infrastructure that allows managers to restrict or provide water to wetlands based on management objectives and water availability (Downard et al. 2014) and also buffers the wetlands to some extent from the decreasing lake levels that made international news in recent years (e.g., Flavelle 2023; Great Salt Lake Collaborative n.d.). However, inter- and intra-annual fluctuations remain the norm despite this infrastructure (Fig. 2a,b). Furthermore, although the Great Salt Lake is a saline lake, the water control infrastructure allows for some flushing of salts from the wetlands to maintain mildly brackish conditions (despite the effects of high salinity due to past flooding; Downard et al. 2014; Null and Wurtsbaugh 2020).

Utah Lake, the third largest lake west of the Mississippi, also provides essential habitat for wildlife and abundant recreational opportunities (Richards 2019;Utah Lake Authority 2024a). As opposed to Farmington Bay, Utah Lake wetlands are largely continuous with the lake itself but at the mercy of lake levels that fluctuate greatly across years due to site evaporation from the shallow lake (maximum depth ~ 3 m) and upstream diversions from the inflowing rivers (Richards 2019; Zanazzi et al. 2020; Utah Lake Authority 2024b). The Utah Lake shoreline (and thus water available for wetlands) can shift long distances (potentially 50–100 s of meters) each year. Shoreline fluctuations are natural in such a shallow lake yet have become more extreme due to climate change (shifts in precipitation, increasing temperatures; Richards 2019). Large changes in water inflows from the Provo, Spanish Fork, and American Fork Rivers also occur inter- and intra-annually due to upstream dam operations on the Deer Creek and Jordanelle Reservoirs and the legal use of Utah Lake as a reservoir (Richards 2019). Such fluctuations can leave Utah Lake wetlands “high and dry” (Fig. 2c) or deeply inundated (Fig. 2d).

At both locations, a collaboration of various state and local agencies has been removing *P. australis* and other invasive species from the area since 2014 (Young and Kettenring 2020; Utah Division of Forestry, Fire and State Lands 2022) to improve habitat diversity for wildlife and public access (Utah Lake Authority 2024a). Both sites have a history of yearly fall aerial or spot spraying of *P. australis* with glyphosate or imazapyr following best management practices (Rohal et al. 2016; 2019a,b; Kettenring et al. 2020). Sites were selected to have minimal reestablishment of *P. australis* at the start of the field experiment (May 2022) and < 2.5 cm of standing water to ensure enough light and oxygen for germination and seedling survival.

### Seed Collection, Testing and Dormancy Break

All seeds were collected from the Great Salt Lake Watershed during the 2021 growing season following the methods of Robinson et al. (2024b). Tetrazolium testing was conducted to determine the viability of each species, which was used to calculate the pure live seed (PLS = viability x purity x bulk seed mass) rate for seeding (AOSA and SCST 2010; Robinson et al. 2024c). Seeds of most species were cold, moist stratified in wet silica sand and peat moss at 2℃ for at least 15 weeks to break physiological dormancy (Tarsa et al. 2022b; Robinson et al. 2024a). However, the *Bolboschoenus maritimus* seed lot was split in half and scarified for 24 or 48 h in a 3% bleach solution to account for intraspecific variation in seed coat thickness and related physiological dormancy (Kettenring 2016; Marty and Kettenring 2017). The intent of the dormancy breaking treatments of the entire seed lot was to ensure that the seeds were ready to germinate (i.e., non-dormant) in the field upon seeding.

### Experimental Seeding and Data Collection

Seeds were sown by hand in 1 m^2^ plots surrounded by mesh fabric to prevent loss of seeds out of the plots by natural water movement (Fig. 1b). Seeding occurred in late May 2022 at Utah Lake and early July 2022 at Farmington Bay. The mid-summer Farmington Bay seeding was later than planned due to the inability to access the originally intended field site. At Farmington Bay, water was briefly released in early August from the neighboring impoundment to provide water to the experiment (Fig. 2). Data were collected until mid-September in 2022 (which represented about 16 weeks for Utah Lake and 11 weeks for Farmington Bay in the first year). For the second growing season (2023), Farmington Bay was monitored from mid-July to mid-September (~ 9 weeks). The site could not be accessed until mid-July 2023 due to deep flooding. Utah Lake was not monitored during 2023 due to excessive flooding that made the site inaccessible for the entirety of the growing season.

Data collection began about two weeks following seeding to allow time for germination. Total percent canopy cover and individual percent canopy cover for each species present in the plot were collected biweekly at each location as follows: < 1%, 1–10%, 11–20%, 21–30%, 31–40%, 41–50%, 51–60%, 61–70%, 71–80%, 81–90%, 91–99%, > 99%. Wells were installed at each block to measure water depth. Depth was measured using a ruler from the surface of the ground to the surface of the groundwater within the ~ 2 m deep wells.

### Statistical Analyses

All statistical analyses for this experiment were conducted using R statistical software (v4.2.1; R Core Team 2022). Data from each site and each year were analyzed separately using generalized linear mixed effects models to assess the effects of the treatments on native and invasive plant final cover using the *glmmTMB* package (v.1.1.5; Brooks et al. 2017). Final cover estimates were converted to continuous proportions using the midpoint of the cover class. Final cover was analyzed using the beta distribution or the log-normal distribution, depending on model fit, as described below. For analyses using the beta distribution, all 0 values were converted to one half the smallest recorded value and all 1 values were converted to 0.995 to fit within the [0,1] bounded distribution. All treatments with values above 100% cover were analyzed using the log-normal distribution. For each site, multiple mixed effects models were used to determine the effects of treatments. Response of total native, invasive, and seeded plant cover for each functional group was tested using native functional group identity (five levels) and native density (two levels) as fixed effects, as well as block (six levels) as a random effect. We also conducted a Dunnett’s test in association with each model to determine whether final cover differed between any of the treatments and the control. Seeded plant cover was analyzed by adding together the seeded species for each functional group. We were unable to fit models to the data for the annual forb, rush, or perennial forb functional groups at the Farmington Bay site for 2022, the grass or rush functional groups at the Utah Lake site for 2022, or any seeded species for the Farmington Bay site for 2023. Model fit was estimated using plot residuals from the *DHARMa* package (v0.4.6; Hartig 2022). Treatment differences were tested using the *anova()* function from the *car* package (v3.1.0; Fox and Weisberg 2019). Pairwise comparisons were calculated using the Tukey HSD method at α = 0.10 through the *emmeans* package (v.1.8.1.1; Lenth 2022). The α was chosen to increase power and reduce the chance of type II error due to low sample size (Day and Quinn 1989). The *multcomp* package (v1.4.20; Hothorn et al. 2008) was used to calculate compact letter displays for the plots. Language to describe the compact letter displays on graphs was based on Piepho (2018). Although there are no discrete cutoffs in significance testing, we are using the language of Muff et al. (2022) to describe the results of the statistical analyses.

## Results

### Passively Recolonizing Plant Communities Differed at the Two Sites (Questions 1, 4)

**Fig. 3:**
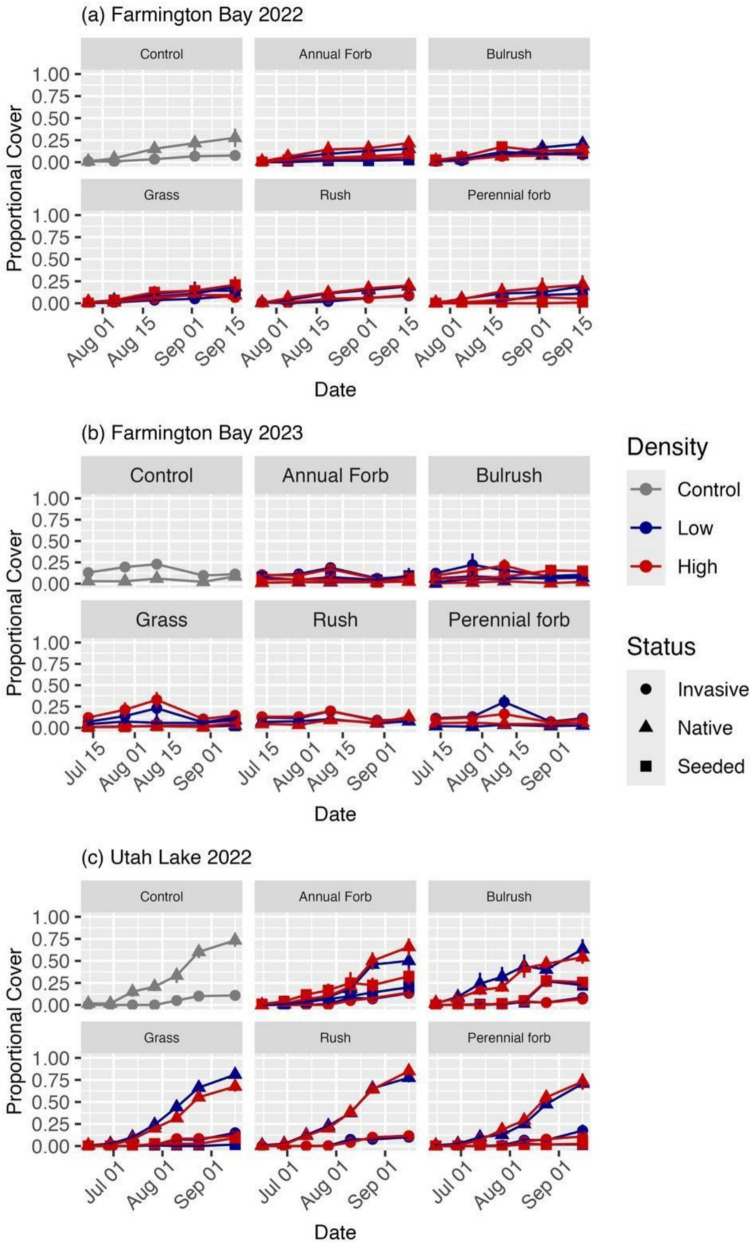
Proportional cover for all invasive, native, and seeded species over time for each seed mix functional group at each native seeding density at Farmington Bay during (**a**) 2022 and (**b**) 2023, and at (**c**) Utah Lake during 2022. Invasive and native refer to the invasion status of species emerging from the seed bank. Seeded refers to species that were seeded in the plot. Raw data are shown (*n* = 6). Error bars represent standard errors

Passively recolonizing plant communities differed by year at Farmington Bay. During 2022, the plant community consisted mostly of the native species *Chenopodium* spp., *Leptochloa fusca*, and *D. spicata*, and the invasive species *Typha* spp. with very few seeded or other invasive species present (Fig. 3a; 4a). However, during 2023, the plant community switched to mostly the invasive species *P. australis* and *Typha* spp. and the native species *Rumex maritimus*, with very few other seeded or unseeded native species (Fig. 3b; 4b). Although *D. spicata* and *R. maritimus* were seeded in this experiment, their presence in all plots, including the unseeded controls, shows that they also emerged independent of our seeding efforts. Although plant composition changed, cumulative plant cover remained low (< 33%) between the two years (Fig. 3a,b).

**Fig. 4:**
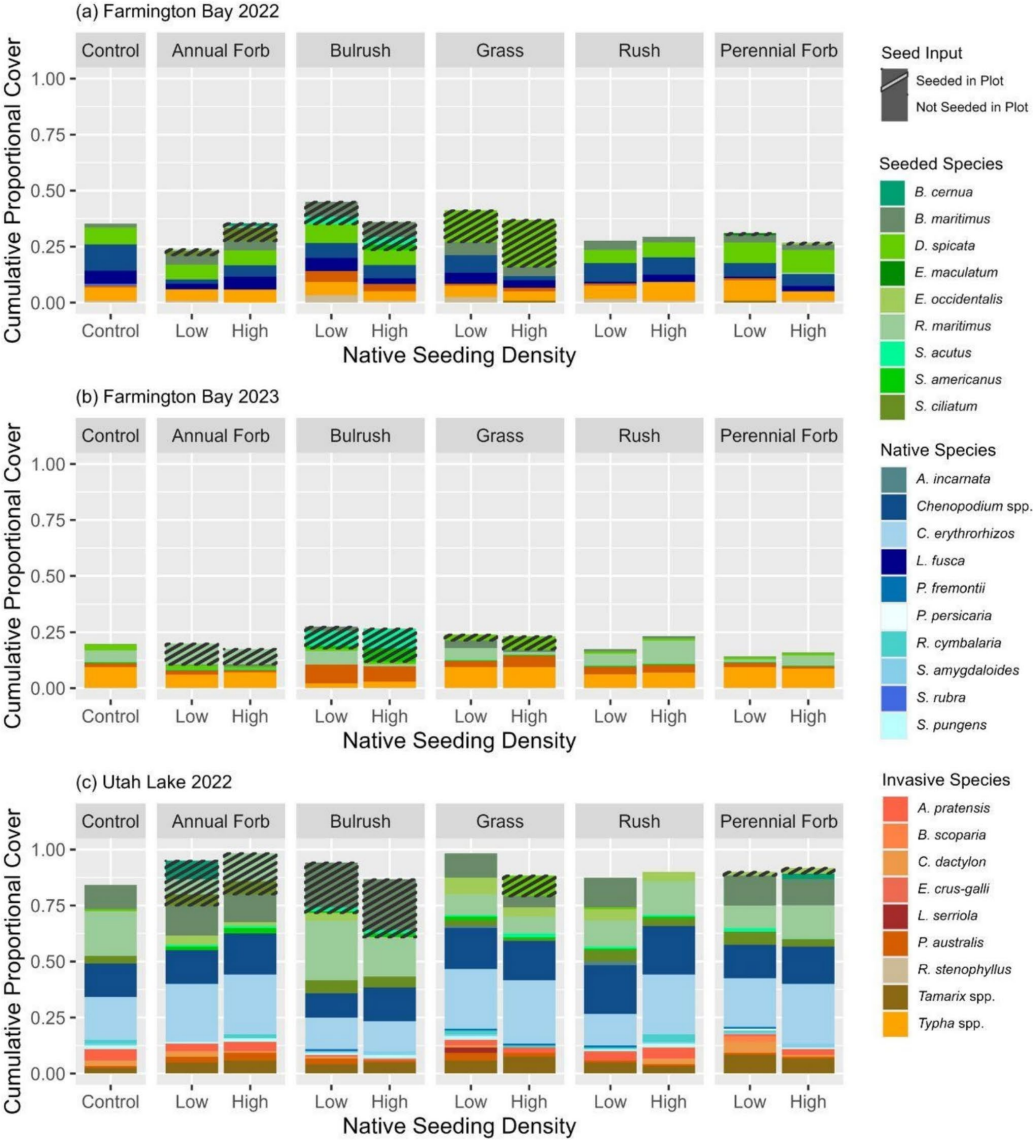
Cumulative proportional cover of all seeded native, unseeded native, and invasive species present for each seed mix functional group at each level of native seeding density at Farmington Bay during (**a**) 2022 and (**b**) 2023, and (**c**) at Utah Lake during 2022 compared to the cumulative proportional cover of all species found in the unseeded control plots (far left). Species are labeled as “seeded species” if they were seeded in any plot. Columns are striped if the species was seeded within the plot to differentiate whether “seeded species” were seeded within the given plot or emerged from the seedbank. Raw data are shown (*n* = 6)

The plant community at Utah Lake in 2022 consisted mostly of the native species *B. maritimus*, *Chenopodium* spp., *Cyperus erythrorhizos, R. maritimus*, and one invasive species (*Tamarix* spp.; Fig. 4c)*.* Although *B. maritimus* and *R. maritimus* were seeded, their presence in every plot suggests they also emerged from the seed bank. Utah Lake had more species and a higher plant cover than Farmington Bay in 2022 (Fig. 3a,c). Although there was some overlap in unseeded species (e.g., *B. maritimus*, *Chenopodium* spp., *D. spicata*), there were more unseeded species present at Utah Lake than at Farmington Bay (Fig. 4a,c).

### Seeding had Minimal Effect and Differed Between Sites (Questions 2, 3, 4)

**Fig. 5:**
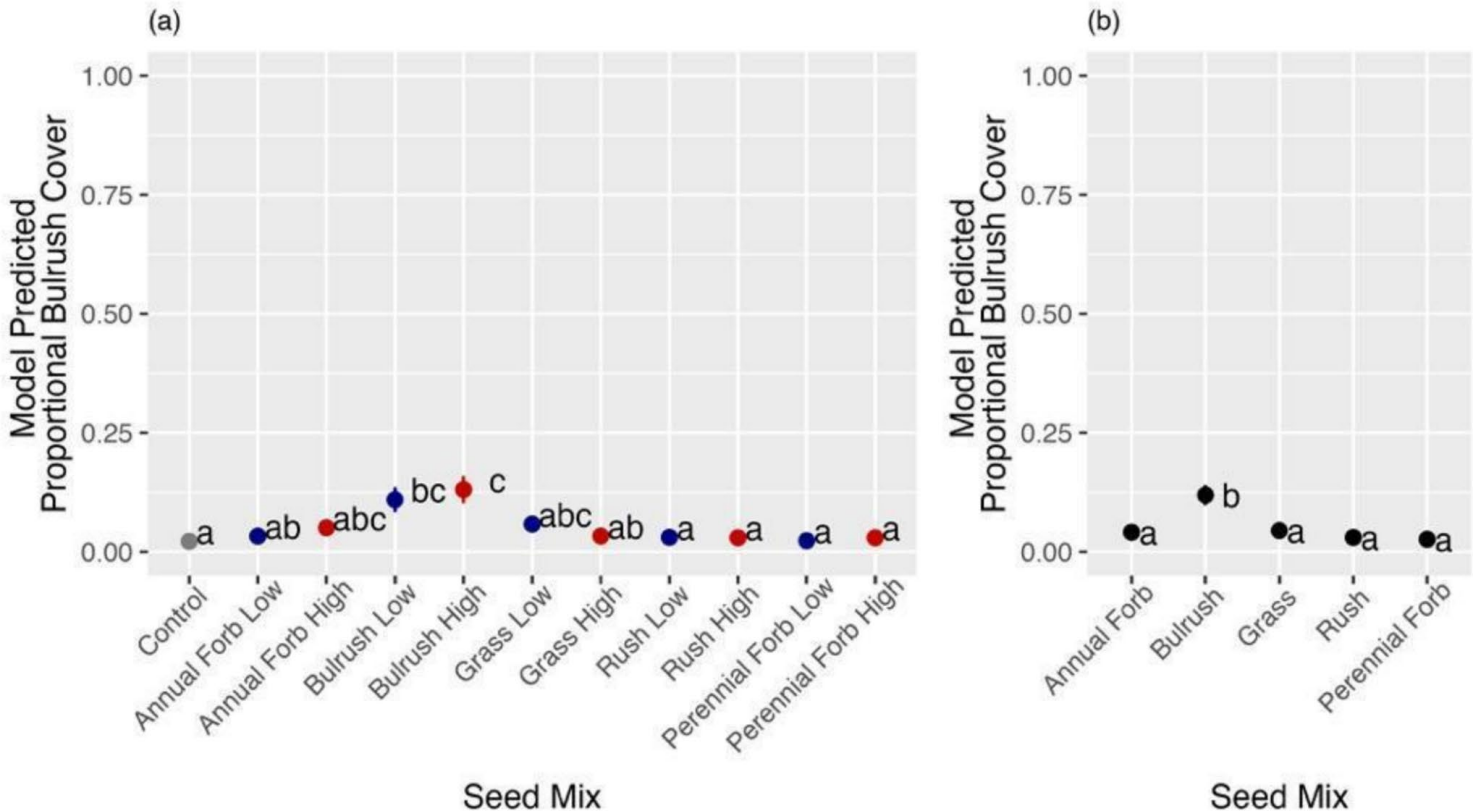
**a** Comparison of proportional final bulrush functional group cover for each seed mix at each density to the unseeded control during 2022 at Farmington Bay. **b** The effect of seed mix functional group on proportional final bulrush functional group cover during 2022 at Farmington Bay. Means followed by a common letter show no evidence of a difference by the Tukey HSD method at α = 0.10. Model means are shown for (**a**) pairwise comparisons of each treatment plot to the control in a Dunnett’s test and (**b**) the GLMM of between-treatment effects for the model of final bulrush functional group cover as a function of seed mix functional group and native seeding density. Error bars represent standard errors

Seeding had little effect on plant cover as neither final native (combined seeded and unseeded) nor invasive cover varied appreciably between treatments or vs. the control for either location or growing season (Tables S1–S6). For seeded native cover, the bulrush functional group during 2022 at Farmington Bay showed evidence of being positively affected by seeding (Tables 3, S7; Fig. 5). Although we found evidence of differences between other groups at both locations during 2022, the results suggest that they were not true effects of seeding because there was no evidence of the treatments differing from the control (Tables S8-S10) or the evidence of differences was not related to the relevant treatment (Table S11).

**Table 3:** (a) Pairwise comparison of each treatment plot to the control in a Dunnett’s test and (b) ANODEV table of between-treatment effects for the model of final bulrush functional group cover as a function of seed mix functional group and native seeding density for 2022 at Farmington Bay

**(a) Contrast**	**Ratio**	**SE**	**P-value**
Perennial forb x Low – Control	1.06	0.56	1.00
Perennial forb x High – Control	1.35	0.70	0.98
Rush x Low – Control	1.39	0.72	0.97
Rush x High – Control	1.35	0.70	0.98
Grass x Low – Control	2.74	1.35	0.27
Grass x High – Control	1.52	0.79	0.94
Bulrush x Low – Control	5.47	2.50	< 0.01
Bulrush x High – Control	6.68	3.02	< 0.001
Annual forb x Low – Control	1.50	0.78	0.94
Annual forb x High – Control	2.36	1.17	0.44
**(b) Model**	***Χ***^2^	***Df***	***Pr(***> ***c***^2^)
Group	36.2	4	< 0.0001
Density	0.2	1	0.70
Group x Density	2.9	4	0.58

**Fig. 6:**
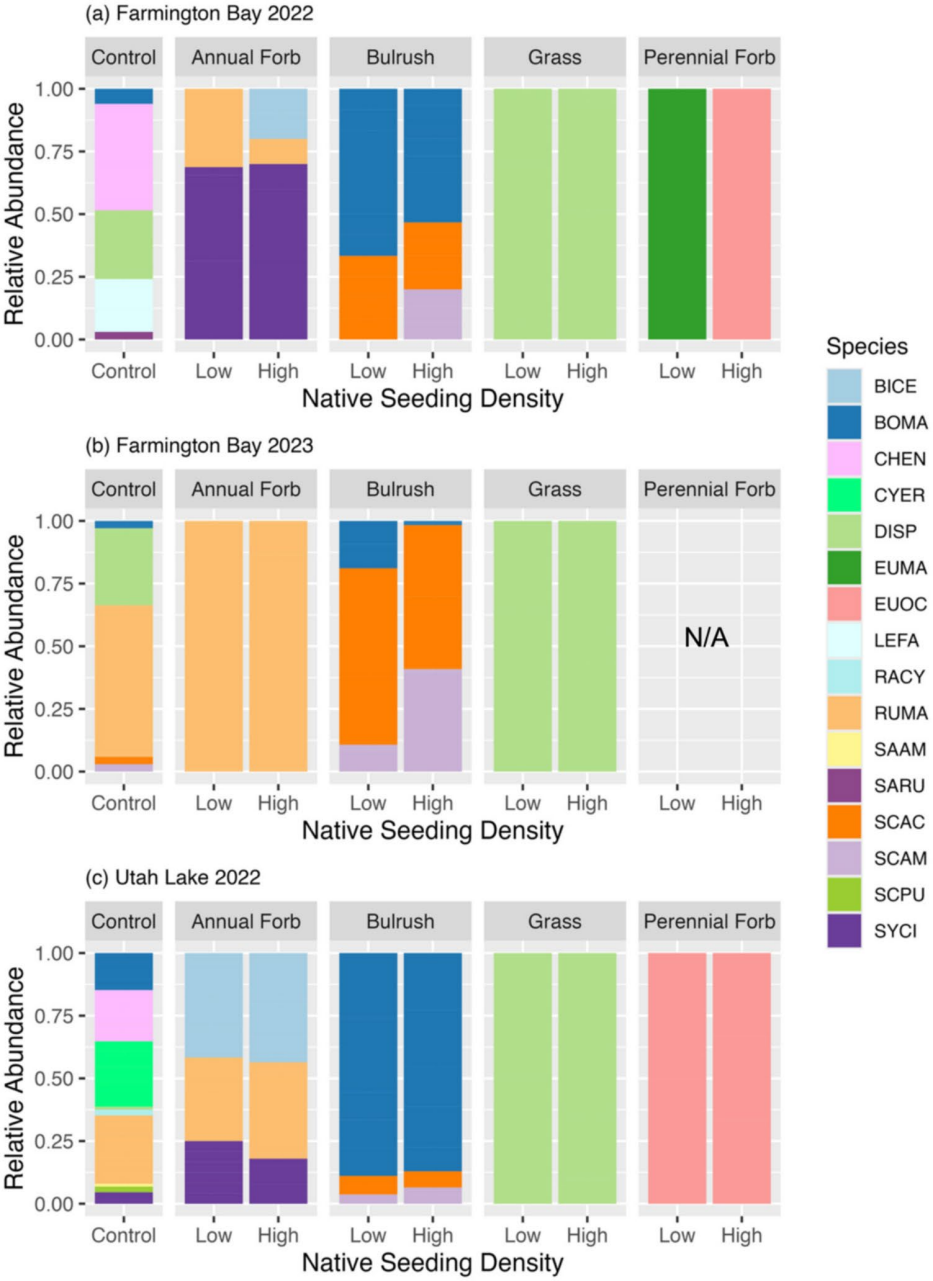
Relative abundance of seeded species cover for each seed mix functional group at each level of native seeding density at Farmington Bay during (**a**) 2022 and (**b**) 2023, and (**c**) at Utah Lake during 2022 compared to the relative abundance of native species cover found in the unseeded control plots (far left). No perennial forbs grew in (**b**) 2023. Species are listed as four letter abbreviations (first two letters of the genus and first two letters of the species epithet). Raw data are shown (n = 6)

**Fig. 7:**
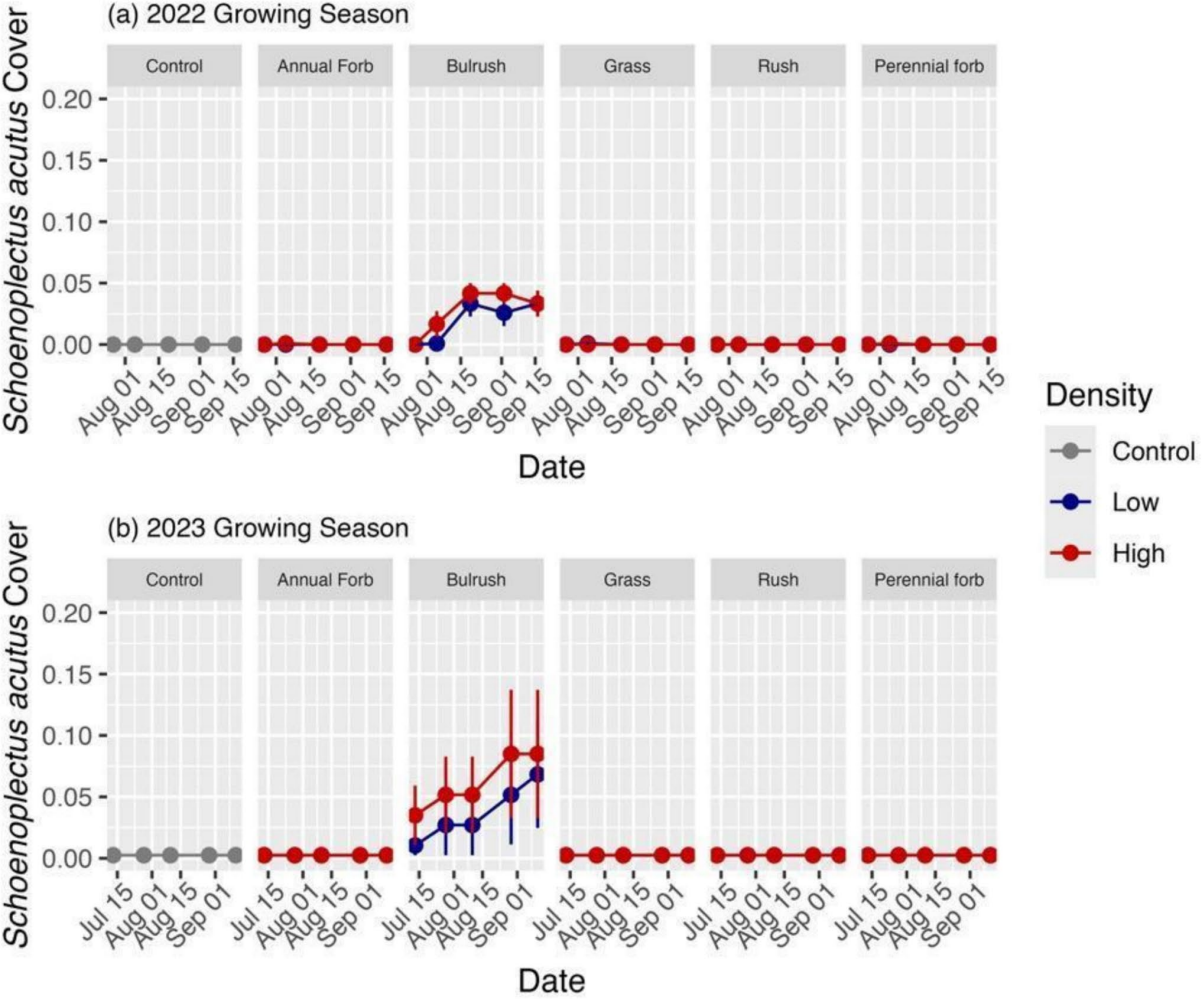
Proportional cover of *Schoenoplectus acutus* over time for each seed mix functional group at each native seeding density during (**a**) 2022 and (**b**) 2023 at Farmington Bay. Raw data are shown (n = 6). Error bars represent standard errors

At Farmington Bay, seeded plant composition differed by year with more seeded species and a higher cumulative proportional cover present in 2022 (Fig. 4a,b). Only three species (*B. maritimus*, *D. spicata*, *S. acutus*) were present both years in any substantial amount (> 15% cover relative to their cover in the control plots; Fig. 6a,b). *Schoenoplectus acutus* also showed substantial growth patterns in line with restoration goals, expanding from ~ 5% cover in 2022 at Farmington Bay up to ~ 13% cover in one of the higher seeding density plots in 2023 (Fig. 7).

Seeded cover and composition also differed between sites in 2022, where it was generally higher at Utah Lake than Farmington Bay. Interestingly, almost all species that germinated at Farmington Bay during 2022 were also present at Utah Lake; however, they differed by relative abundance of cover and the plots in which they grew (Fig. 4a,c; 6a,c).

### Plots were Dry During the 2022 Growing Season but Flooded During the 2023 Growing Season (Question 4)

At Farmington Bay, groundwater fell deep below the soil surface (18–76 cm below) for the majority of 2022 (Fig. 2a). Water briefly reached the soil surface in early August after managers released water from the neighboring impoundment to provide water to the experiment; however, the effects of releasing this water did not persist. In 2023, all blocks had standing water (8–25 cm above) for the majority of the growing season, only falling below the soil surface in mid-September (4–13 cm below; Fig. 2b). Sites could not be accessed before the first sampling date in mid-July due to deep flooding that made the site inaccessible. At Utah Lake, groundwater was hovering around the soil surface across all blocks at the start of the 2022 growing season and gradually fell well below the soil surface (5–85 cm below; Fig. 2c). Water depth at Utah Lake was not assessed in 2023 because plots were inaccessible (> 1.5 m; Fig. 2d).

## Discussion

Restoration of native wetland communities is a common goal following invasive plant management (Adams et al. 2021), however, uncertainties remain about the value of seeding given its logistical difficulties and low chance of survival (Kettenring and Tarsa 2020; Larson et al. 2015). In this experiment, we examined the effects of passive recolonization and seeding on wetland community cover and composition at two locations within the same watershed over two growing seasons. We identified four key findings from this research. First, passive recolonization alone was insufficient to increase native plant cover and reduce the number of invasive species. The two sites differed greatly in their passive recolonization, but neither site achieved restoration goals related to increasing natives and reducing invasives. Second, we found indications that the effects of passive recolonization and seeding on cover and composition of the seeded, unseeded native, and invasive communities varied by site despite geographic proximity. Although in the same watershed, the two field sites differed in final cover and overall plant composition. We hypothesize that these site-specific differences are related to differences in salinity and hydrology at the two locations. Third, surprisingly and unfortunately, it seems that extremes in water depth during the two study years (drought in 2022 and flooding in 2023) overwhelmed the seeding treatments to drive plant community composition. Yet, these hydrologic extremes, which are becoming increasingly common due to climate change and human water withdrawals (Wurtsbaugh et al. 2017; IPCC 2023), provided an opportunity to evaluate the recovery potential of the various functional groups. In turn, we identified a subset of species that are of interest to managers (*B. maritimus* and *S. acutus* from the bulrush functional group and *D. spicata* from the grass functional group) that were able to survive the extreme weather conditions of both growing seasons at Farmington Bay. These findings underscore the importance of not relying on passive recovery but instead seeding diverse seed mixes as a bet-hedging strategy. As unpredictable extreme weather events and hydrologic fluctuations become more common in wetlands, there will be a greater need for bet-hedging strategies to promote native growth regardless of the abiotic conditions that arise.

### Passive Recolonization was Insufficient to Achieve Restoration Goals Following Invasive Species Management

Although passive recolonization following invasive plant management may sometimes achieve wetland restoration goals, revegetation is often a necessary step to increase native plant cover and decrease the chance of invasive species reinvasion (Adams and Galatowitsch 2008; Kettenring and Adams 2011; Tarsa et al. 2022a). Although the two sites in the present study differed greatly in passive recolonization, neither of the two locations resulted in both high native species cover and low invasive species cover, typical restoration goals following invasive species management. At Farmington Bay, overall native plant cover remained low across both growing seasons, likely explained by the fact that seed bank emergence can be very low following multi-year invasive plant management (Rohal et al. 2021). Large stands of invasive species such as *P. australis*, which have been treated at Farmington Bay for over a decade, block the dispersal of seeds of nearby native plant communities, which can have long-lasting effects on the seed bank (Elsey-Quirk and Leck 2021). Furthermore, these large stands can leave behind litter (which was anecdotally noted by study authors at the site), which can also block seeds from having sufficient contact with the soil (Cranney 2016). Restoration efforts in locations with such histories will likely require additional modification during restoration, such as seeding and litter removal (Rohal et al. 2016; Tarsa et al 2022b).

Farmington Bay also exhibited potential to return to an invasive dominated state, likely due to the extreme weather that affected conditions at the site. Invasive species are successful in part because they can survive broader and more extreme conditions than native species (Zedler and Kercher 2004; Hovick et al. 2023), making it important to identify native species traits that contribute to tolerance or resilience to a range of conditions in wetlands (Moor et al. 2017). In fact, during the second growing season, the research plots at Farmington Bay were dominated by invasive species, suggesting that these species could better survive the extreme hydrologic conditions, while many of the native species could not. This was true even though all species that emerged at Farmington Bay during the second growing season (seeded, unseeded native, and invasive) were all flood tolerant species, as shown by their wetland indicator status of facultative, facultative wetland, and obligate. This finding is consistent with those of Byun et al. (2020), who, in a study of species and functional diversity effects on biotic resistance in a freshwater wetland, found that *P. australis* had increased invasion success in wetlands following flooding.

Utah Lake had much higher total plant cover in the passively recovering community than Farmington Bay, showing potential that passive recolonization might be a viable strategy for increasing native cover. However, the plant community still included a large diversity of invasive species, suggesting a high likelihood of the community reverting to be invasive-dominated in the future. Although seeding in any context is useful for increasing native plant cover (Tarsa et al. 2022a), it is especially important to seed when invasive propagule pressure is high (Adams and Galatowitsch 2008)—a fact that is particularly important for Utah wetlands, which have been shown to have unusually high *P. australis* propagule pressure (Rohal et al. 2021). Furthermore, efforts to shape wetland plant communities to be more resilient and resistant to invasion are particularly important in areas prone to extreme weather conditions (Hovick et al. 2023), such as the flooding seen in Utah Lake during 2023. Although we were unable to monitor for a third season to determine the effects of the flooding on the plant community, we would expect invasive species to benefit from the flooding (Zedler and Kercher 2004; Byun et al. 2020), as they did at Farmington Bay.

### The Effects of Passive Recolonization and Seeding on Cover and Composition of the Seeded, Unseeded Native, and Invasive Communities Varied by Site

Even in sites that are geographically close and experiencing the same climate, different restoration outcomes can result from small changes in biotic and abiotic conditions unique to sites (Young et al. 2015; Fried et al. 2018). In the present study, the two sites differed substantially in the cover and composition of the unseeded native, seeded native, and invasive community. Plant community differences could be attributed to many biotic and abiotic factors, but salinity or hydrology are likely large drivers. The brackish wetland of Farmington Bay contained a higher cover of salt-tolerant species like *D. spicata* and *S. rubra* (i.e., reflecting the fact that species have different salinity tolerances; Tootoonchi et al. 2023) and a plant community composition with fewer species (i.e., species were filtered out due to inadequate salt tolerances). The recolonizing wetland plant communities subject to the more benign freshwater conditions of Utah Lake had a greater number of unseeded native species than Farmington Bay but a lower proportion of halophytic species*.*

These plant community differences were likely also driven by the hydrology of the two sites, specifically long-term lake-level fluctuation. Lakes that fluctuate yearly have greater diversity, as differences in abiotic conditions allow for more niches and increased germination from the seed bank (Keddy and Reznicek 1986; Wilcox and Nichols 2008), a fact that may explain why more species emerged from the background community at Utah Lake than Farmington Bay. Although both sites experienced drought in 2022, Utah Lake is known to have yearly fluctuations in lake level due to evaporation and upstream water diversions (Richards 2019). Although Farmington Bay also experiences water-level fluctuations (as was seen in this experiment), managers at Farmington Bay have coarse control over water level to lessen the effects of water-level fluctuations on restoration outcomes (Downard et al. 2017), as seen when managers released water into the experimental area in early August 2022.

### Water Depth Appeared to Overwhelm Seeding Treatments

Drought can be a major constraint to successful restoration, as water availability can lead to rapid mortality of the more vulnerable seed and seedling stages of plants relative to more established plants (Doherty and Zedler 2015; Buisson et al. 2021; Hebert 2022). On the other hand, flooding can drive wetland community assembly because different species germinate and survive at different water depths with associated variations in light, temperature, and oxygen (Spence 1982; Webb et al. 2012). In the present study, hydrologic extremes, both severe drought and prolonged and, at times, deep flooding, appeared to overwhelm the functional group and seeding density treatments. At Farmington Bay, most of the seeded species never emerged, and the majority that germinated in the first growing season were absent by the second season. Although emergent species can survive a range of hydrologic conditions, the set of conditions under which seeds can germinate and grow is narrower (Rosbakh et al. 2020). One germination study from a wetland seed bank found that growth was reduced across the entire seed bank when water was 6 cm below the soil surface and greatly reduced when water was 2 cm or greater above the soil surface (Fraser and Karnezis 2005). In the present experiment, water levels at Farmington Bay were 8–76 cm below the soil surface in 2022 and 8–25 cm above the soil surface in 2023, suggesting that few seedlings were able to germinate and survive in 2022 and any that were able to emerge in 2022 were likely unable to establish in 2023. At Utah Lake, the plots were flooded by > 1.5 m of water in year 2 over the entire growing season, so the plots themselves were not identifiable, and certainly, the seedlings would not have survived if they had withstood the drought in year 1.

The extreme weather events presented an opportunity to identify species that survived these extremes, suggesting they may be good focal species for wetland restoration. We found that three species (*B. maritimus* and *S. acutus* from the bulrush functional group and *D. spicata* from the grass functional group) have the potential to survive the hydrologic extremes present in the field that will become more common at both sites as a result of climate change (Wurtsbaugh et al. 2017; Richards 2019; IPCC 2023). Managers value the three species for their habitat benefits (Downard et al. 2017; Rohal et al. 2018). The species also represent a range of hydrologic tolerances, likely contributing to their resistance or resilience to hydrologic extremes. Bulrushes, in particular, showed great potential for growing under varied environmental conditions as they were the only species for which seeding had a measurable effect on native cover in the study. This finding is consistent with other studies that found that *B. maritimus* can tolerate water levels ranging from drought to flooding, as well as highly alkaline and salty soils (Downard et al. 2017; Houde et al. 2024). The seeds can also germinate even when below the water surface (Clevering 1995). Furthermore, *S. acutus* was the only seeded species to increase over time during both growing seasons. Although *S. acutus* typically prefers flooding (Stewart and Kantrud 1972; Downard et al. 2017), these results suggest that it has the potential to establish and spread under broad hydrologic conditions. Finally, *D. spicata* was also able to survive both years of extreme weather at Farmington Bay, aligning with previous studies that found *D. spicata* to survive both regular inundation (Stalter et al. 2022) and extreme drought (McKee et al. 2006).

The ability to compare hydrologic data to plant growth outcomes is complicated to evaluate and has rarely been tested (Brudvig et al. 2017; Sueltenfuss and Cooper 2019). Methodological limitations (e.g., linking hydrology to delayed plant responses; collecting plant and hydrology data on compatible spatial and temporal scales) are particularly problematic given that the inability to manage hydrology adequately for plant communities is a major reason that restoration projects fail (Bohnen and Galatowitsch 2005; Galatowitsch and Bohnen 2021). The water level data we collected (at the block level intended to describe site conditions) does not allow statistical comparison with plot-level plant data. However, given that field experiments are notoriously poor at elucidating drivers of plant community assembly due to many uncontrollable factors, our findings emphasize the need for controlled experiments (e.g., in the greenhouse) to more precisely identify drivers of salinity, hydrology, and their interactions on plant community assembly in this system.

### Diverse Seed Mixes can Act as a Bet-Hedging Strategy for Restoration Success

Given the difficulty of predicting environmental conditions within wetlands (Bohnen and Galatowitsch 2005), particularly with increasing hydrologic extremes due to climate change and human water withdrawals (Wurtsbaugh et al. 2017; IPCC 2023), wetland restoration should focus on incorporating diversity on multiple levels of a restoration project through the use of a bet-hedging strategy (also called a portfolio effect; Schindler et al. 2015). Given that extreme weather events are unpredictable and restoration projects often take place on small geographic scales, portfolio effects can be particularly important for increasing resilience to environmental extremes in restoration projects to combat this increased volatility (Zabin et al. 2022). The results of this experiment exemplify how seeding species from different functional groups and hydrologic tolerances can serve as a form of bet-hedging. Although we did not design this experiment to test the effects of bet-hedging, seeding a variety of species (i.e., across the functional group identity treatments) resulted in some native growth during both hydrologic extremes, something that may not have occurred if only flood-tolerant or drought-tolerant species had been introduced. Seed mixes should include a wide variety of species from different functional groups to ensure that restoration goals can be met regardless of weather and site conditions and to avoid biotic homogenization, which can lead to a loss of diversity and ecosystem functioning (Holl et al. 2022; Luong et al. 2023). Furthermore, seeding a variety of species allowed us to ensure native growth at both locations even without precise knowledge of the future abiotic conditions.

A bet-hedging strategy can be further enhanced with reseeding sites over multiple years and breaking dormancy in only a subset of the seed mix to increase the probability of aligning seed and seedling abiotic requirements with favorable environmental conditions for establishment in future years (Doherty and Zedler 2015; Rader et al. 2022; Zabin et al. 2022). Interestingly, we broke dormancy for all seed mixes under the presumption that the seeds would be sown at the optimal time for germination and seedling establishment. Yet, we did not foresee the hydrologic extremes facing the restoration sites post-seeding. Treating a fraction, like half, of the seed lot (as a bet-hedging strategy) would have been an additional, but not insurmountable, logistical hurdle that might be feasible for others to implement in wetland seedings, thereby building up the seed bank for the future when environmental conditions are better suited for seeds (Evans and Dennehy 2005), especially given that annual weather is a determinant of restoration success (Copeland et al. 2024). In addition, re-seeding sites over multiple years may also be required in a future with greater environmental extremes (Groves and Brudvig 2019; Kettenring and Tarsa 2020). Given the difficulty of predicting abiotic conditions at a site within a short temporal scale, it is important to incorporate bet-hedging to ensure restoration success regardless of conditions. This research was a useful step in exemplifying the importance of this strategy for the many restoration projects where year-to-year hydrologic extremes may ultimately drive outcomes.

## Supplementary Information

Below is the link to the electronic supplementary material.

Supplementary file 1 (DOCX 691 KB)

## Data Availability

Data are available on GitHub at https://github.com/WetlandEcologyLab/Grad_Feldman_Elana.git
