# Supplementary material for: Bet Hedging to Aid Seed-Based Wetland Restoration Under Hydrologic Extremes
Source: Wetlands (Wilmington). 2025 May 22;45(5):52. doi: 10.1007/s13157-025-01935-7 (PMC12098206; doi:10.1007/s13157-025-01935-7)
Supplement: Supplementary file 1 — (DOCX 691 KB) [file 13157_2025_1935_MOESM1_ESM.docx]

**Table S1** (a) Pairwise comparison of each treatment plot to the control in a Dunnett’s test and (b) ANODEV table of between-treatment effects for the model of final native cover as a function of seed mix functional group and native seeding density for the 2022 growing season at Farmington Bay. There was no evidence for an effect of group when multiple comparisons were made using the Tukey HSD method at α = 0.10

| **(a) Contrast** | **Ratio** | **SE** | ***P*-value** |
| --- | --- | --- | --- |
| Perennial forb 𝗑 Low – Control | -0.08 | 0.06 | 0.76 |
| Perennial forb 𝗑 High – Control | -0.06 | 0.06 | 0.89 |
| Rush 𝗑 Low – Control | -0.09 | 0.06 | 0.67 |
| Rush 𝗑 High – Control | -0.08 | 0.06 | 0.76 |
| Grass 𝗑 Low – Control | 0.05 | 0.06 | 0.93 |
| Grass 𝗑 High – Control | 0.03 | 0.06 | 0.99 |
| Bulrush 𝗑 Low – Control | 0.03 | 0.06 | 0.99 |
| Bulrush 𝗑 High – Control | -0.0008 | 0.06 | 1.00 |
| Annual forb 𝗑 Low – Control | -0.10 | 0.06 | 0.54 |
| Annual forb 𝗑 High – Control | 0.03 | 0.06 | 0.99 |
| **(b) Model** | ***Χ^2^*** | ***Df*** | ***Pr(>c^2^)*** |
| Group | 10.7 | 4 | 0.03 |
| Density | 0.6 | 1 | 0.44 |
| Group 𝗑 Density | 5.2 | 4 | 0.27 |

**Table S2** (a) Pairwise comparison of each treatment plot to the control in a Dunnett’s test and (b) ANODEV table of between-treatment effects for the model of final native cover as a function of seed mix functional group and native seeding density for the 2023 growing season at Farmington Bay

| **(a) Contrast** | **Ratio** | **SE** | ***P*-value** |
| --- | --- | --- | --- |
| Perennial forb 𝗑 Low – Control | 0.35 | 0.20 | 0.39 |
| Perennial forb 𝗑 High – Control | 0.41 | 0.24 | 0.58 |
| Rush 𝗑 Low – Control | 0.70 | 0.41 | 0.97 |
| Rush 𝗑 High – Control | 0.94 | 0.55 | 0.99 |
| Grass 𝗑 Low – Control | 1.20 | 0.70 | 0.99 |
| Grass 𝗑 High – Control | 0.80 | 0.47 | 0.99 |
| Bulrush 𝗑 Low – Control | 1.11 | 0.65 | 0.99 |
| Bulrush 𝗑 High – Control | 1.56 | 0.91 | 0.95 |
| Annual forb 𝗑 Low – Control | 0.60 | 0.35 | 0.91 |
| Annual forb 𝗑 High – Control | 0.93 | 0.54 | 0.99 |
| **(b) Model** | ***Χ^2^*** | ***Df*** | ***Pr(>c^2^)*** |
| Group | 4.1 | 4 | 0.39 |
| Density | 0.1 | 1 | 0.74 |
| Group 𝗑 Density | 0.9 | 4 | 0.93 |

**Table S3** (a) Pairwise comparison of each treatment plot to the control in a Dunnett’s test and (b) ANODEV table of between-treatment effects for the model of final native cover as a function of seed mix functional group and native seeding density for the 2022 growing season at Utah Lake

| **(a) Contrast** | **Ratio** | **SE** | ***P*-value** |
| --- | --- | --- | --- |
| Perennial forb 𝗑 Low – Control | 1.00 | 0.12 | 1.00 |
| Perennial forb 𝗑 High – Control | 1.09 | 0.13 | 0.96 |
| Rush 𝗑 Low – Control | 1.07 | 0.13 | 0.98 |
| Rush 𝗑 High – Control | 1.30 | 0.16 | 0.23 |
| Grass 𝗑 Low – Control | 1.21 | 0.15 | 0.56 |
| Grass 𝗑 High – Control | 1.06 | 0.13 | 0.99 |
| Bulrush 𝗑 Low – Control | 1.17 | 0.14 | 0.74 |
| Bulrush 𝗑 High – Control | 1.09 | 0.13 | 0.96 |
| Annual forb 𝗑 Low – Control | 1.03 | 0.13 | 1.00 |
| Annual forb 𝗑 High – Control | 1.33 | 0.16 | 0.17 |
| **(b) Model** | ***Χ^2^*** | ***Df*** | ***Pr(>c^2^)*** |
| Group | 2.4 | 4 | 0.67 |
| Density | 1.4 | 1 | 0.23 |
| Group 𝗑 Density | 6.7 | 4 | 0.15 |

**Table S4** (a) Pairwise comparison of each treatment plot to the control in a Dunnett’s test and (b) ANODEV table of between-treatment effects for the model of final invasive cover as a function of seed mix functional group and native seeding density for 2022 at Farmington Bay

| **(a) Contrast** | **Ratio** | **SE** | ***P*-value** |
| --- | --- | --- | --- |
| Perennial forb 𝗑 Low – Control | 1.52 | 0.47 | 0.67 |
| Perennial forb 𝗑 High – Control | 0.78 | 0.27 | 0.95 |
| Rush 𝗑 Low – Control | 1.13 | 0.36 | 1.00 |
| Rush 𝗑 High – Control | 1.22 | 0.39 | 0.97 |
| Grass 𝗑 Low – Control | 1.03 | 0.33 | 1.00 |
| Grass 𝗑 High – Control | 0.97 | 0.32 | 1.00 |
| Bulrush 𝗑 Low – Control | 2.06 | 0.61 | 0.12 |
| Bulrush 𝗑 High – Control | 1.12 | 0.36 | 1.00 |
| Annual forb 𝗑 Low – Control | 0.80 | 0.27 | 0.97 |
| Annual forb 𝗑 High – Control | 0.64 | 0.23 | 0.74 |
| **(b) Model** | ***Χ^2^*** | ***Df*** | ***Pr(>c^2^)*** |
| Group | 11.9 | 4 | 0.02 |
| Density | 4.0 | 1 | 0.05 |
| Group 𝗑 Density | 4.8 | 4 | 0.31 |

**Table S5** (a) Pairwise comparison of each treatment plot to the control in a Dunnett’s test and (b) ANODEV table of between-treatment effects for the model of final invasive cover as a function of seed mix functional group and native seeding density for the 2023 growing season at Farmington Bay

| **(a) Contrast** | **Ratio** | **SE** | ***P*-value** |
| --- | --- | --- | --- |
| Perennial forb 𝗑 Low – Control | 0.95 | 0.35 | 0.99 |
| Perennial forb 𝗑 High – Control | 0.80 | 0.30 | 0.98 |
| Rush 𝗑 Low – Control | 0.79 | 0.30 | 0.97 |
| Rush 𝗑 High – Control | 0.77 | 0.29 | 0.96 |
| Grass 𝗑 Low – Control | 1.02 | 0.37 | 0.99 |
| Grass 𝗑 High – Control | 1.26 | 0.45 | 0.97 |
| Bulrush 𝗑 Low – Control | 0.79 | 0.30 | 0.97 |
| Bulrush 𝗑 High – Control | 0.58 | 0.23 | 0.69 |
| Annual forb 𝗑 Low – Control | 0.65 | 0.25 | 0.82 |
| Annual forb 𝗑 High – Control | 0.63 | 0.24 | 0.78 |
| **(b) Model** | ***Χ^2^*** | ***Df*** | ***Pr(>c^2^)*** |
| Group | 5.2 | 4 | 0.27 |
| Density | 0.1 | 1 | 0.76 |
| Group 𝗑 Density | 0.9 | 4 | 0.92 |

**Table S6** (a) Pairwise comparison of each treatment plot to the control in a Dunnett’s test and (b) ANODEV table of between-treatment effects for the model of final invasive cover as a function of seed mix functional group and native seeding density for the 2022 growing season at Utah Lake

| **(a) Contrast** | **Ratio** | **SE** | ***P*-value** |
| --- | --- | --- | --- |
| Perennial forb 𝗑 Low – Control | 0.27 | 0.40 | 0.96 |
| Perennial forb 𝗑 High – Control | -0.13 | 0.42 | 1.00 |
| Rush 𝗑 Low – Control | -0.22 | 0.43 | 0.99 |
| Rush 𝗑 High – Control | -0.18 | 0.42 | 0.99 |
| Grass 𝗑 Low – Control | 0.05 | 0.40 | 1.00 |
| Grass 𝗑 High – Control | -0.18 | 0.42 | 0.99 |
| Bulrush 𝗑 Low – Control | -0.28 | 0.43 | 0.97 |
| Bulrush 𝗑 High – Control | -0.72 | 0.45 | 0.51 |
| Annual forb 𝗑 Low – Control | 0.14 | 0.40 | 1.00 |
| Annual forb 𝗑 High – Control | -0.28 | 0.42 | 0.97 |
| **(b) Model** | ***Χ^2^*** | ***Df*** | ***Pr(>c^2^)*** |
| Group | 3.5 | 4 | 0.48 |
| Density | 2.2 | 1 | 0.14 |
| Group 𝗑 Density | 0.8 | 4 | 0.94 |

**Table S7** Pairwise comparison of functional groups for the model of final bulrush functional group cover as a function of seed mix functional group and native seeding density for the 2022 growing season at Farmington Bay. Calculated using the Tukey HSD method at α = 0.10

| **Contrast** | **Estimate** | **SE** | ***P*-value** |
| --- | --- | --- | --- |
| Perennial forb – Rush | -0.14 | 0.36 | 0.99 |
| Perennial forb – Grass | -0.55 | 0.35 | 0.54 |
| Perennial forb – Bulrush | -1.61 | 0.32 | <0.001 |
| Perennial forb – Annual forb | -0.46 | 0.35 | 0.69 |
| Rush – Grass | -0.41 | 0.35 | 0.77 |
| Rush – Bulrush | -1.47 | 0.31 | <0.001 |
| Rush – Annual forb | -0.32 | 0.35 | 0.88 |
| Grass – Bulrush | -1.07 | 0.30 | <0.01 |
| Grass – Annual forb | 0.08 | 0.33 | 1.00 |
| Bulrush – Annual forb | 1.15 | 0.30 | <0.01 |

**Table S8** (a) Pairwise comparison of each treatment plot to the control in a Dunnett’s test and (b) ANODEV table of between-treatment effects for the model of final grass functional group cover as a function of seed mix functional group and native seeding density for the 2022 growing season at Farmington Bay

| **(a) Contrast** | **Ratio** | **SE** | ***P*-value** |
| --- | --- | --- | --- |
| Perennial forb 𝗑 Low – Control | 3.03 | 2.02 | 0.49 |
| Perennial forb 𝗑 High – Control | 0.68 | 0.45 | 0.98 |
| Rush 𝗑 Low – Control | 0.84 | 0.56 | 1.00 |
| Rush 𝗑 High – Control | 0.61 | 0.41 | 0.95 |
| Grass 𝗑 Low – Control | 1.26 | 0.84 | 1.00 |
| Grass 𝗑 High – Control | 5.07 | 3.39 | 0.13 |
| Bulrush 𝗑 Low – Control | 1.67 | 1.11 | 0.95 |
| Bulrush 𝗑 High – Control | 0.54 | 0.36 | 0.90 |
| Annual forb 𝗑 Low – Control | 0.54 | 0.36 | 0.90 |
| Annual forb 𝗑 High – Control | 0.54 | 0.36 | 0.90 |
| **(b) Model** | ***Χ^2^*** | ***Df*** | ***Pr(>c^2^)*** |
| Group | 12.0 | 4 | 0.02 |
| Density | 1.0 | 1 | 0.32 |
| Group 𝗑 Density | 10.5 | 4 | 0.03 |

**Table S9** (a) Pairwise comparison of each treatment plot to the control in a Dunnett’s test and (b) ANODEV table of between-treatment effects for the model of final bulrush functional group cover as a function of seed mix functional group and native seeding density for the 2022 growing season at Utah Lake

| **(a) Contrast** | **Ratio** | **SE** | ***P*-value** |
| --- | --- | --- | --- |
| Perennial forb 𝗑 Low – Control | 1.68 | 0.72 | 0.76 |
| Perennial forb 𝗑 High – Control | 1.29 | 0.57 | 0.98 |
| Rush 𝗑 Low – Control | 1.72 | 0.74 | 0.73 |
| Rush 𝗑 High – Control | 2.00 | 0.84 | 0.49 |
| Grass 𝗑 Low – Control | 1.02 | 0.46 | 1.00 |
| Grass 𝗑 High – Control | 0.80 | 0.37 | 0.99 |
| Bulrush 𝗑 Low – Control | 2.79 | 1.15 | 0.11 |
| Bulrush 𝗑 High – Control | 2.27 | 0.94 | 0.30 |
| Annual forb 𝗑 Low – Control | 1.90 | 0.80 | 0.57 |
| Annual forb 𝗑 High – Control | 1.25 | 0.55 | 0.99 |
| **(b) Model** | ***Χ^2^*** | ***Df*** | ***Pr(>c^2^)*** |
| Group | 12.5 | 4 | 0.01 |
| Density | 1.1 | 1 | 0.30 |
| Group 𝗑 Density | 1.0 | 4 | 0.91 |

**Table S10** (a) Pairwise comparison of each treatment plot to the control in a Dunnett’s test and (b) ANODEV table of between-treatment effects for the model of final perennial forb functional group cover as a function of seed mix functional group and native seeding density for the 2022 growing season at Utah Lake

| **(a) Contrast** | **Ratio** | **SE** | ***P*-value** |
| --- | --- | --- | --- |
| Perennial forb 𝗑 Low – Control | 0.49 | 0.25 | 0.65 |
| Perennial forb 𝗑 High – Control | 0.99 | 0.47 | 1.00 |
| Rush 𝗑 Low – Control | 0.56 | 0.28 | 0.78 |
| Rush 𝗑 High – Control | 0.80 | 0.38 | 0.99 |
| Grass 𝗑 Low – Control | 0.57 | 0.28 | 0.81 |
| Grass 𝗑 High – Control | 0.38 | 0.19 | 0.35 |
| Bulrush 𝗑 Low – Control | 1.34 | 0.63 | 0.97 |
| Bulrush 𝗑 High – Control | 0.72 | 0.35 | 0.97 |
| Annual forb 𝗑 Low – Control | 0.96 | 0.45 | 1.00 |
| Annual forb 𝗑 High – Control | 1.51 | 0.70 | 0.91 |
| **(b) Model** | ***Χ^2^*** | ***Df*** | ***Pr(>c^2^)*** |
| Group | 9.0 | 4 | 0.06 |
| Density | 0.1 | 1 | 0.71 |
| Group 𝗑 Density | 5.2 | 4 | 0.26 |

**Table S11** (a) Pairwise comparison of each treatment plot to the control in a Dunnett’s test and (b) ANODEV table of between-treatment effects for the model of final annual forb functional group cover as a function of seed mix functional group and native seeding density for the 2022 growing season at Utah Lake

| **(a) Contrast** | **Ratio** | **SE** | ***P*-value** |
| --- | --- | --- | --- |
| Perennial forb 𝗑 Low – Control | 2.71 | 1.98 | 0.67 |
| Perennial forb 𝗑 High – Control | 4.47 | 3.27 | 0.27 |
| Rush 𝗑 Low – Control | 8.85 | 6.47 | 0.03 |
| Rush 𝗑 High – Control | 5.37 | 3.93 | 0.17 |
| Grass 𝗑 Low – Control | 17.51 | 12.80 | <0.01 |
| Grass 𝗑 High – Control | 12.14 | 8.87 | 0.01 |
| Bulrush 𝗑 Low – Control | 7.37 | 5.39 | 0.06 |
| Bulrush 𝗑 High – Control | 1.00 | 0.73 | 1.00 |
| Annual forb 𝗑 Low – Control | 5.37 | 3.93 | 0.17 |
| Annual forb 𝗑 High – Control | 2.71 | 1.98 | 0.67 |
| **(b) Model** | ***Χ^2^*** | ***Df*** | ***Pr(>c^2^)*** |
| Group | 11.4 | 4 | 0.02 |
| Density | 2.4 | 1 | 0.12 |
| Group 𝗑 Density | 3.9 | 4 | 0.42 |
